# *T* Gene Mutation Leads to Short Tail in Sheep via Premature AER Degeneration: Single-Cell Evidence from Embryos

**DOI:** 10.3390/ani16111748

**Published:** 2026-06-05

**Authors:** Hong Su, Yanyan Yang, Yongchun Zuo, Yongli Song, Daqing Wang, Min Zhang, Guifang Cao

**Affiliations:** 1 Key Laboratory of Animal Embryo and Development Engineering of Universities of Higher Learning, Hohhot 010011, China; 2 College of Agriculture and Animal Husbandry Science and Technology, Ningxia Vocational and Technical College for Minorities, Wuzhong 751100, China; 3 Inner Mongolia Academy of Agricultural & Animal Husbandry Sciences, Hohhot 010031, China; 4 College of Life Sciences, Inner Mongolia University, Hohhot 010021, China; 5 College of Veterinary Medicine, Inner Mongolia Agricultural University, Hohhot 010010, China

**Keywords:** *T* gene, sheep, short tail, apical ectodermal ridge, single-cell RNA sequencing, embryonic development, genetic mutation, precision breeding

## Abstract

Hulunbuir short-tailed sheep (HSTS) and Hu sheep (HS) exhibit distinct tail phenotypes linked to ecological adaptation, with HSTS carrying a loss-of-function mutation (c.G334T) in the *T* gene while HS retain the wild-type allele. However, the cellular and molecular mechanisms underlying *T*-mediated tail development remain unclear. Here, we performed single-cell RNA sequencing on HSTS and HS embryos at embryonic days 16 and 19 (E16 and E19), complemented by cross-species validation using a CRISPR/Cas9 mouse model carrying the same mutation. We identified 12 cell types in E16 HSTS and E16 HS embryos, and 15 cell types in E19 HSTS and E19 HS embryos and found that the *MDK_ITGA6+ITGB1* ligand–receptor pair consistently mediated core intercellular communication. The *MDK_ITGA6+ITGB1* axis mediates intercellular communication critical for tail bud formation; BMP activation and FGF repression disrupt AER survival, leading to tail shortening. Developmental trajectories showed a shift from early progenitor states at E16 to terminal differentiation at E19. Crucially, HSTS embryos showed transcriptomic signatures consistent with premature AER regression. The *T* mutation showed transcriptomic signatures of increased BMP pathway activity and reduced *FGF8* expression, which may disrupt AER survival and contribute to the short-tail phenotype. In the mouse model, mutant *T* expression was reduced, and expression dynamics of *WNT5B* and *FGF8* were perturbed, corroborating the sheep findings; however, homozygous *T* mutation causes embryonic lethality in mice but not in sheep, indicating species-specific differences. This study provides single-cell transcriptomic evidence linking the *T* c.G334T mutation to premature AER regression in sheep, complemented by cross-species validation in a CRISPR/Cas9 mouse model, offering new insights into the cellular mechanisms of tail development and may provide a basis for future investigations into tail-related breeding markers, pending experimental validation. These changes are associated with AER maintenance and tail outgrowth.

## Simple Summary

Tail length in sheep is an important trait influenced by both genetics and environmental adaptation. Hulunbuir short-tailed sheep have naturally short tails that help them survive in cold climates, whereas Hu sheep have a relatively longer fat-tail compared to HSTS, which may aid in thermoregulation in warm and humid environments. Previous research identified a mutation in the *T* gene associated with the short-tail phenotype, but the underlying mechanism remained unclear. In this study, we used single-cell technology to compare embryonic development between these two sheep breeds. We discovered that the *T* gene mutation causes premature degeneration of a structure called the apical ectodermal ridge, which is essential for tail formation. This degeneration results from a disrupted balance between two signaling pathways—one that promotes cell survival and another that induces cell death. Our findings explain how a single gene mutation shapes tail development and provide valuable insights for breeding programs aimed at improving sheep adaptability to diverse environments.

## 1. Introduction

Hulunbuir short-tailed sheep (HSTS) and Hu sheep (HS) represent two distinct genetic resources of indigenous Chinese sheep breeds, with their tail phenotype divergence reflecting long-term environmental adaptation. HSTS have been reared in Inner Mongolia for nearly a century and are characterized by strong environmental resilience and short thick fat tails (8–14 cm in length). In contrast, HS display a moderately elongated fat-tail (compared to typical short-fat-tailed breeds), which facilitates thermoregulation under hot and humid conditions, and are known for rapid growth, high reproductive performance, and strong tolerance to heat and humidity [1,2].

*T* (*Brachyury*) is a conserved transcription factor essential for mesoderm and notochord development. In sheep, HSTS carry a missense mutation (c.G334T, p.Gly112Cys) in the DNA-binding domain, while HS have the wild-type allele. According to studies by Guifang Cao et al., the c.G334T mutation is associated with a reduced expression level in embryos, with expression patterns closely correlated with the short-tailed phenotype [3]. Functional validation in mice showed that heterozygotes exhibit 20–30% tail shortening, whereas homozygotes die during early embryogenesis due to notochordal defects [4].

However, the precise cellular and molecular mechanisms through which the *T* mutation regulates tail development remain unknown.

With the rapid advancement of biotechnology, single-cell RNA sequencing (scRNA-seq) has emerged as a fundamental approach for dissecting cellular heterogeneity and transcriptional dynamics. By capturing the transcriptome of individual cells, scRNA-seq overcomes the limitations of bulk sequencing, providing unprecedented insights into developmental biology, disease mechanisms, and cellular regulation [5]. This technique has been widely applied to the construction of embryonic transcriptional atlases in species including mice, humans, non-human primates, zebrafish, and pigs, as well as to the reconstruction of organ developmental trajectories [6,7,8,9,10,11,12,13].

We hypothesized that the c.G334T mutation disrupts AER maintenance by altering the balance between BMP and FGF signaling, leading to premature AER regression and a short tail. This study constructs single-cell transcriptional atlases of HSTS and HS embryos to investigate how the c.G334T mutation is associated with the short-tailed phenotype, and validates key findings in a CRISPR/Cas9 mouse model. This work provides a theoretical framework for understanding the developmental genetic basis of environmentally adaptive traits and may inform future studies on genetic markers for tail-related traits, although direct breeding applications require further validation.

## 2. Materials and Methods

The study compared HSTS (mutant) and HS (wild-type) sheep at embryonic days 16 and 19 using single-cell RNA sequencing, with key findings validated by RT-qPCR in both sheep and a CRISPR/Cas9 mouse model.

### 2.1. Ethical Statement

All sheep and mice experimental procedures and protocols were approved and authorized by the animal care and use committee of the Inner Mongolia Agricultural University (Inner Mongolia Autonomous Region, China) in this study (Permit No. SYXK 2021-0022).

### 2.2. Animal Management

Ten HSTS and ten HS ewes were synchronized using progesterone-containing PRID vaginal sponges (SYNCRITE-45, Animal Health Supplies, Melbourne, Australia) for 12 days. At sponge withdrawal, each ewe received an intramuscular injection of 330 IU pregnant mare serum gonadotropin (PMSG; SanSheng Biological Technology, Ningbo, China). Artificial insemination was performed 38 h later. On embryonic days 16 and 19 (E16 and E19), the ewes were sacrificed, and the uteri (with attached ovaries) were collected. Embryos were flushed from the uteri using prewarmed phosphate-buffered saline (PBS, 37 °C; Gibco, New York, NY, USA) and preserved in MACS^®^ Tissue Storage Solution (Miltenyi, Bergisch Gladbach, Germany).

In total, one HSTS embryo at E19, three HS embryos at E16, and one HS embryo at E19 were collected and stored at 4 °C in preservation solution, followed by scRNA-seq within 24 h. Single-cell transcriptomic data for HSTS embryos at E16 were retrieved from the Gene Expression Omnibus (GEO) database (GSE185233). Specific-pathogen-free (SPF) grade C57BL/6 mice were used in this study for functional validation experiments.

For the scRNA-seq analysis, one HSTS embryo at E19, three HS embryos at E16, and one HS embryo at E19 were collected and processed. The scRNA-seq data for the E16 HSTS embryo were retrieved from the GEO database (GSE185233). Thus, the number of biological replicates per group is as follows: E16 HSTS, *n* = 3; E16 HS, *n* = 3; E19 HSTS, *n* = 1; E19 HS, *n* = 1. Randomization was not applicable because samples were selected solely based on developmental stage (E16/E19) and genotype (HSTS vs. HS); all embryos were processed identically. Samples were selected solely based on developmental stage (E16/E19) and genotype (HSTS vs. HS).

Mice were housed under SPF conditions with a 12 h light/dark cycle and ad libitum access to food and water. For validation experiments, at least three wild-type and three heterozygous mutant embryos were collected per time point (E7.5–E12.5) from three independent litters.

### 2.3. scRNA-Seq

Sheep embryos were enzymatically dissociated in calc-Mg-free HBSS containing collagenase II (Thermo Fisher Scientific, Waltham, MA, USA. 17101015, 100 U/μL) and collagenase IV (Thermo Fisher Scientific, 17104019, 100 U/μL) at 37 °C. Based on preliminary optimization, a digestion time of 15 min was selected. Following digestion, cell suspensions were passed through a 70 μm strainer and centrifuged at 400 *g* and 4 °C for 5 min. After the supernatant was removed, the pellet was resuspended in red blood cell lysis buffer (Miltenyi) and incubated on ice for 5 min to lyse erythrocytes. The reaction was quenched with PBS+2%FBS. Centrifuged at 400 *g* at 4 °C for 5 min. Then discard the supernatant and resuspend, followed by centrifugation at 300 *g* for 5 min at 4 °C. The cell pellet was resuspended and filtered through a 40 μm strainer. Cell concentration and viability were determined using a Countstar Rigel S5 automated counter (Ruiyu Biotech Co., Ltd., Shanghai, China).

Approximately 17,000 cells were loaded onto the 10× Chromium Controller (10× Genomics, Pleasanton, CA, USA), and libraries were prepared using the Single-Cell 3′Reagent Kit v3.1 according to the manufacturer’s protocol. GEM-Reverse Transcription (GEM-RT) was performed on a thermal cycler with the following program: 53 °C for 45 min, 85 °C for 5 min, and hold at 4 °C.

After reverse transcription and cell barcoding, emulsions were broken, and cDNA was isolated and purified using Cleanup Mix containing DynaBeads and SPRIselect reagent (Thermo Fisher Scientific). Purified cDNA was PCR-amplified, and quality was assessed using the Agilent Bioanalyzer (Agilent Technologies, Santa Clara, CA, USA). For RNA-seq library construction, amplified cDNA underwent fragmentation, end repair, dual size selection, PCR amplification with sample-specific indexing primers, and a final dual size selection.

Libraries were purified, quality-controlled, and sequenced on an Illumina NovaSeq 6000 platform (Illumina, Inc., San Diego, CA, USA) using 150 bp paired-end reads.

### 2.4. Data Preprocessing

Experimental design overview. This study compared two sheep breeds (HSTS mutant vs. HS wild-type) at two developmental stages (E16 and E19). scRNA-seq was performed on one E19 HSTS embryo, three E16 HS embryos, and one E19 HS embryo; scRNA-seq data for E16 HSTS were obtained from GEO (GSE185233, three biological replicates). All libraries were prepared using the 10× Genomics 3‘ v3.1 kit and sequenced on an Illumina NovaSeq 6000 platform. Technical variability was minimized by processing all samples in parallel. Key findings were validated by RT-qPCR using biological triplicates in both sheep and mouse.

Raw sequencing reads in FASTQ format were processed using the sheep reference genome (Ovis aries Ovis_aries_genome_v1.0) and analyzed with the Cell Ranger pipeline (v6.1.2) to generate gene expression matrices. Downstream analyses were performed in R (v4.1.2) using the Seurat package (v4.1.1) [14]. Gene expression and metadata from different samples were integrated using custom R scripts, and a combined Seurat object was created for further processing.

Data filtering criteria. The percentage of mitochondrial transcripts (percent.mt) was calculated for each cell and stored as metadata. Low-quality cells were filtered out using the following thresholds: number of detected genes (nFeature_RNA) between 500 and 6000, total UMI counts (nCount_RNA) between 1000 and 30,000, and mitochondrial percentage < 5% for high-quality cells. After initial filtering, cells with more than 200 genes and less than 10% mitochondrial content were retained. Specifically, cells with nFeature_RNA < 200 or >6000, nCount_RNA < 1000 or >30,000, or percent.mt > 10% were excluded. These criteria were based on visual inspection of QC metrics and standard practices in scRNA-seq analysis.

Normalization and imputation. Normalization was performed using the NormalizeData function (normalization.method = “LogNormalize,” scale.factor = 10,000), followed by log-transformation. Missing values were imputed using the MAGIC algorithm (magic v3.0.0) [15], with parameters set to genes = “all_genes.”. MAGIC imputation was applied because the *T* gene showed severe dropout in raw data, preventing its detection. After imputation, *T* expression became consistent with RT-qPCR validation, indicating that imputation helped recover genuine biological signals. All other conclusions were also verified on raw data where possible.

Highly variable gene selection. Highly variable genes were identified with the FindVariableFeatures function (selection.method = “vst,” nfeatures = 2000). The number of highly variable genes (2000) is the default value in Seurat and is widely used in scRNA-seq studies to balance sensitivity and noise.

Scaling and regression. The ScaleData function (vars.to.regress = “percent.mt”) was used to assess the effect of “percent.mt” and perform regression, followed by dimensionality reduction and clustering using the scaled and centered residuals.

Principal component analysis. To reduce the dimensionality of the dataset, the RunPCA function was applied to the linearly transformed and scaled data generated by ScaleData with default parameters. ElbowPlot and DimHeatmap evaluation was conducted to determine the first 25 principal components (PCs) as input for the FindNeighbors() function. These 25 PCs explained >80% of the total variance, and the ElbowPlot showed that the variance contribution plateaued after 25 PCs, justifying their retention.

Batch effect correction with Harmony. To integrate the public dataset (GSE185233) and our in-house sequenced samples, we used Harmony (v0.1.0) [16] for batch correction. Harmony was applied based on the sample source to remove technical differences while preserving biological heterogeneity. Cells were projected into a shared low-dimensional embedding space, allowing clustering by cell type rather than dataset-specific conditions. After Harmony correction, neighbor graph construction (FindNeighbors), clustering analysis (FindClusters, resolution = 0.2), and UMAP visualization (RunUMAP) were performed using the first 25 dimensions (dims = 1:25). All other parameters were set to default values. The corrected Harmony embeddings were used for all downstream analyses, including dimensionality reduction, clustering, and differential expression.

Clustering and visualization. Clustering was then performed using the FindClusters function across different resolution values (tested range: 0.1–0.4, including 0.1, 0.2, 0.25, 0.3, 0.4). Resolution 0.25 was selected because it produced biologically interpretable clusters where known cell types (e.g., apical ectodermal ridge, notochord, somites) were clearly separated. Lower resolutions (e.g., 0.1–0.2) merged distinct cell types, while higher resolutions (≥0.3) artificially split biologically similar populations. Thus, a final resolution of 0.25 was chosen. Dimensionality reduction was visualized in two-dimensional space using Uniform Manifold Approximation and Projection (UMAP).

Cell-type annotation and differential expression analysis. Cell-type annotation was carried out by comparing known marker genes with combinatorial expression patterns across subpopulations. Cell type annotations were cross-validated using the publicly available sheep embryo scRNA-seq dataset (GSE185233) and the CellMarker 2.0 database. Differentially expressed genes (DEGs) were identified using the FindAllMarkers function in Seurat (only.pos = TRUE), retaining those with *p* < 0.05 (Wilcoxon rank-sum test, default). DEGs were ranked by average log2 fold change (avg_log2FC). For exploratory differential expression analysis, nominal *p*-values are reported without multiple testing correction, as the primary goal was hypothesis generation. All key findings were independently validated by RT-qPCR. Gene Ontology (GO) enrichment analysis and Kyoto Encyclopedia of Genes and Genomes (KEGG) pathway enrichment analysis were performed using the clusterProfiler package (v4.2.2). A complete list of key parameters and software versions used in the scRNA-seq analysis is provided in Table A2.

### 2.5. CellChat Analysis

Cell–cell interaction analysis was performed using the CellChat R package (v2.1.2). All CellChat analyses were performed with a fixed random seed (set.seed(123)) to ensure reproducibility. Default parameters were used unless otherwise specified. A CellChat object was constructed from the Seurat object with the newCellDataSet function. The computeCommunProbPathway function was applied to aggregate all relevant ligand–receptor pairs and calculate pathway-level communication probabilities. Intercellular communication networks were computed using the aggregateNet function, and the number and strength of communications between cell types were visualized using the netVisual_circle function. The netVisual_diffInteraction function was used to visualize pathway-level alterations between samples, and the rankNet function was applied to rank inter-sample pathway differences. Within each sample, the netAnalysis_contribution function was used to identify the contribution of each ligand–receptor pair to overall signaling pathways, enabling visualization of intercellular communication mediated by individual ligand–receptor pairs. The netVisual_bubble function was employed to display key ligand–receptor interactions between cell types and important pathway-associated interactions in both samples. The plotGeneExpression function was used to generate violin plots showing expression differences in signaling genes across cell types and samples. Finally, signaling pathway network centrality scores were calculated and visualized using the netAnalysis_computeCentrality function.

### 2.6. Pseudotime Analysis

Cell–cell trajectory inference was performed using the Monocle R package (v2.22.0) [17]. Pseudotime analysis was performed with set.seed(123). The DDRTree algorithm was run with maxIter = 100 and default parameters. Raw gene expression data from the Seurat object were converted into a CellDataSet object with the newCellDataSet function. Genes differentially expressed across subclusters were used as ordering genes. Developmental trajectories were constructed using the reduceDimensions function (reduction_method = “DDRTree”) to build a minimum spanning tree. Genes differentially expressed along pseudotime and at branch points were subjected to GO and KEGG enrichment analysis using the clusterProfiler3 R package (v4.2.2).

### 2.7. SCENIC Analysis

Cell Single-cell regulatory network inference and clustering were performed using pySCENIC (v0.11.2). Co-expression modules were first derived from expression matrices using the grn parameter. Motif enrichment analysis was then performed with the ctx step, applying a normalized enrichment score (NES) threshold of 2.5 to identify enriched cis-regulatory motifs and candidate target genes. The aucell step was subsequently used to quantify the activity of gene signatures at the single-cell level.

For the transcription factor T, downstream target genes were identified from enriched motif information and visualized in regulatory network diagrams. After normalizing the regulon specificity score (RSS) of *T*, single-cell regulatory network inference was repeated within the limb ectoderm cell population, which displayed higher *T* specificity. The R package igraph (v1.5.1) was used for visualizing *T*-regulated downstream genes within the limb ectoderm.

### 2.8. PCR Amplification

PCR amplification was performed with Green Taq Mix (P131-01, Vazyme, Nanjing, China) to genotype *T* alleles in DNA extracted from hindlimb toes of neonatal mice, all embryonic mouse samples, and both male and female HSTS and HS individuals used in this study. Primer sequences are provided in Table 1.

PCR conditions: initial denaturation at 95 °C for 5 min; 35 cycles of 95 °C for 30 s, 58 °C for 30 s, 72 °C for 45 s; final extension at 72 °C for 5 min.

### 2.9. RT-qPCR Validation

Genomic DNA and total RNA were extracted from HSTS and HS embryos at E16 and E19 using the RNA/DNA isolation kit (DP422, Tiangen, Beijing, China), following the manufacturer’s instructions. Reverse transcription was performed with the HiScript II Q RT SuperMix kit (R223-01, Vazyme, Nanjing, China) using RNA isolated from sheep embryos (E16, E19) and from C57BL/6 mouse embryos (E7-E12) of both wild-type and mutant *T* genotypes. RT-qPCR was carried out with ChamQ Universal SYBR qPCR Master Mix (Q711-02, Vazyme, Nanjing, China) on a CFX96 Real-Time PCR System (Bio-Rad, Hercules, CA, USA). RT-qPCR cycling conditions: 95 °C for 30 s, followed by 40 cycles of 95 °C for 15 s and 60 °C for 30 s, with a melt curve stage (60–95 °C). Melt curve analysis confirmed a single specific product for each primer pair. *GAPDH* served as the internal reference gene. Relative expression levels were calculated using the 2^−ΔΔCT^ method. ΔΔCt was calculated by subtracting the ΔCt of the calibrator sample (wild-type E16 HS for sheep, wild-type E10.5 mouse for mouse) from the ΔCt of each test sample. Each sample was run in technical triplicate. No-template controls (NTC) were included in each run. Data are presented as mean ± SD. Statistical comparisons between wild-type and mutant groups were performed using a two-tailed Student’s *t*-test, with *p* < 0.05 considered significant. Primer sequences are listed in Table 2.

## 3. Results

### 3.1. Analysis of the T Gene c.334 Locus Genotypes

PCR-based genotyping was performed on genomic DNA extracted from one HSTS ram, one HS ram, ten HSTS ewes, and ten HS ewes. Among the 11 HSTS individuals tested, all were homozygous for the c.G334T mutation, and among the 11 HS individuals tested, all were homozygous for the wild-type allele. This genotyping confirmed the genotypes of the animals used for scRNA-seq and RT-qPCR; it does not represent population-level frequencies. Detailed results are presented in Table 3.

### 3.2. Clustering and Identification of Whole-Embryo Samples from E16 and E19 HSTS and HS

**Figure 1:**
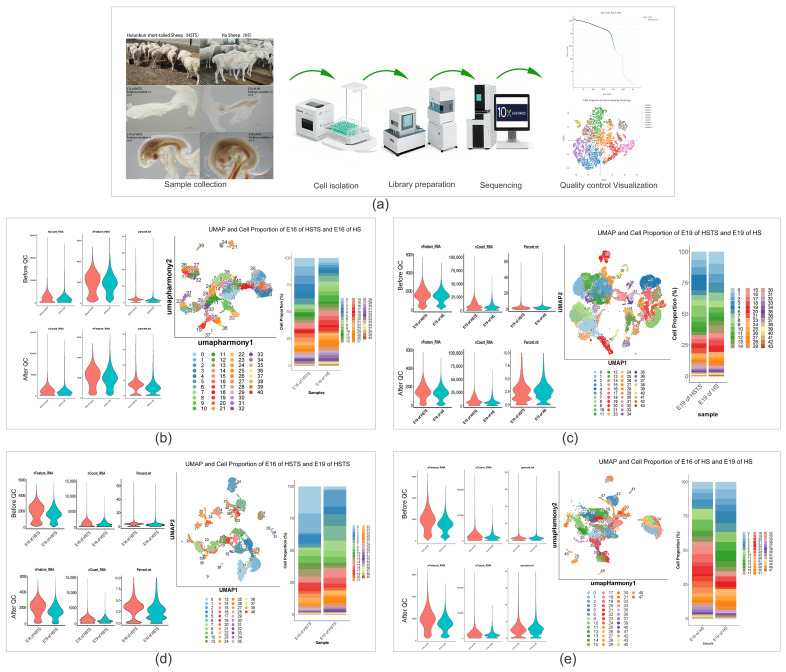
Single-cell transcriptome sequencing of E16 and E19 embryos of HSTS and HS. (**a**) Procedure of single-cell transcription sequencing. (**b**) Quality control of single-cell transcriptome data, UMAP of single-cell transcription sequencing data, and cluster distribution in E16 HSTS and E16 HS groups. (**c**) Quality control of single-cell transcriptome data, UMAP of single-cell transcription sequencing data, and cluster distribution in E19 HSTS and E19 HS groups. (**d**) Quality control of single-cell transcriptome data, UMAP of single-cell transcription sequencing data, and cluster distribution in E16 HSTS and E19 HSTS groups. (**e**) Quality control of single-cell transcriptome data, UMAP of single-cell transcription sequencing data, and cluster distribution in E16 HS and E19 HS groups.

In this study, scRNA-seq was performed on one E19 HSTS embryo, three E16 HS embryos, and one E19 HS embryo. The scRNA-seq dataset of the E16 HSTS embryo was obtained from the GEO database (GSE185233) (Figure 1a). Subsequently, pairwise analyses were conducted across the groups, structured around three key dimensions: (1) Cross-breed comparisons at the same developmental stage (Figure 1b: E16 HSTS vs. E16 HS; Figure 1c: E19 HSTS vs. E19 HS), to identify breed-specific heterogeneity in molecular regulatory networks and dissect divergent regulatory mechanisms; (2) Intra-breed comparisons across developmental stages (Figure 1d: E16 HSTS vs. E19 HSTS; Figure 1e: E16 HS vs. E19 HS), to elucidate the evolution of core developmental modules and reconstruct molecular trajectories of early ovine embryogenesis; (3) Mechanistic dissection of tail phenotype formation, focusing on the cascade effects of *T* gene mutations on tail development, thereby establishing the causal links among genotype, molecular regulation, and phenotype. The number of distinct cell types identified was 12 for E16 HSTS, 12 for E16 HS, 15 for E19 HSTS, and 15 for E19 HS.

For scRNA-seq data quality control, three key parameters were evaluated: the number of detected genes (nFeature_RNA, expected range: 500–6000), the total UMI counts (nCount_RNA, expected range: 1000–30,000), and the proportion of mitochondrial genes (percent.mt, <5%). Both the E16 HSTS and E16 HS samples showed nFeature_RNA values of approximately 2100, nCount_RNA ranging from 5000 to 30,000, and percent.mt around 3%. These QC metrics met the requirements for downstream analyses. Based on the sequencing data, Seurat-based workflows were employed for cell clustering. A total of 41 clusters were identified using UMAP (Figure 1b).

For the E19 HSTS and E19 HS samples, nFeature_RNA values were 1800, nCount_RNA ranged from 5000 to 30,000, and percent.mt was 2.5%. QC results were within acceptable thresholds. Seurat analysis yielded 42 clusters. Clusters containing <10 cells or with a median percent.mt > 10% (Clusters 6 and 22) were excluded from downstream analysis (Figure 1c).

Regarding the E16 vs. E19 HSTS comparison, nFeature_RNA was 2000, nCount_RNA ranged from 10,000 to 50,000, and percent.mt was 2.5–3%. After QC, Seurat analysis identified 37 clusters. Clusters containing <10 cells or with a median percent.mt > 10% (Clusters 4, 11, 18, and 39) were excluded from downstream analysis (Figure 1d).

Similarly, in the E16 vs. E19 HS comparison, nFeature_RNA was 2000, nCount_RNA ranged from 10,000 to 50,000, and percent.mt was 2.5%. QC results met the requirements. Seurat analysis produced 41 clusters. Clusters containing <10 cells or with a median percent.mt > 10% (Clusters 4, 7, 8, 16, 34, 38, and 44) were excluded from downstream analysis (Figure 1e). Additional QC data are provided in Table A1.

**Figure 2:**
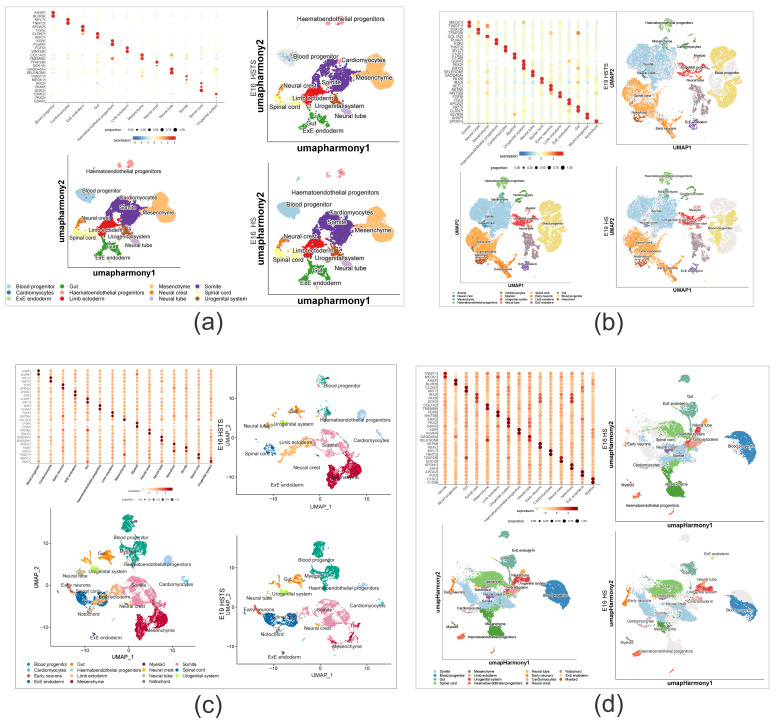
Single-cell transcriptomic atlas of E16 and E19 HSTS and HS embryos. (**a**) Single-cell transcriptional atlas of E16 HSTS and E16 HS. (**b**) Single-cell transcriptional atlas of E19 HSTS and E19 HS. (**c**) Single-cell transcriptional atlas of E16 HSTS and E19 HSTS. (**d**) Single-cell transcriptional atlas of E16 HS and E19 HS.

Cell type annotation of E16 and E19 HSTS and HS embryos was performed by referencing the CellMarker 2.0 database, the GeneCards database, and relevant literature. In vertebrate embryogenesis, distinct cell types are characterized by specific developmental origins and gene expression profiles. Somites (paraxial mesoderm) express *TWIST1* and *MEOX1* [18,19,20]; blood progenitors express *AHSP* and *BLVRB* [21]; neuromesodermal progenitors (NMPs) of spinal cord origin express *IRX3*, *PAX6*, and *SOX2* [22,23]; gut cells express *CLDN7* and *KRT7* [6,24]; haematoendothelial progenitors (EPCs/angioblasts) express *KDR* and *PLVAP* [25,26,27]; the urogenital system (intermediate mesoderm/urogenital ridge) expresses *EMX2*, *PAX2*, and *GDNF* [28,29,30]; extraembryonic endoderm expresses *APOA2* and *FGG* [31,32]; cardiomyocytes express *MYL7* and *TNNT2* [33]; mesenchymal stem cells express *COL1A2* and *TMEM88* [34,35]; the limb ectoderm (corresponding to the apical ectodermal ridge, AER) expresses *FGF8* and *WNT5B* [36,37,38]; neural crest cells (migratory ectoderm-derived populations) express *TFAP2B* and *SOX10* [39]; the neural tube (CNS progenitors) expresses *GADD45A* and *SELENOM* [40,41]; myeloid cells express *C1QC* and *C1QB* [42,43]; early neurons express *NEFM* and *NEFL* [44,45]; and notochord cells express *SPON1* [46] (Figure 2). “The expression patterns of these markers in our data were consistent with those reported in the reference sheep embryo dataset (GSE185233), supporting the reliability of our cell annotations. Annotations were further manually reviewed by two independent researchers to ensure biological plausibility”. The presence of AER cells only in HS at E19, and their absence in HSTS, suggests that the *T* mutation accelerates AER regression, which is likely the cellular basis of the short-tail phenotype.

### 3.3. CellChat Analysis of E16 HSTS vs. E16 HS and E19 HSTS vs. E19 HS Samples

To elucidate the heterogeneity of intercellular communication networks during embryonic development, CellChat (v2.1.2) was employed to analyze cell–cell interactions in E16 HSTS vs. E16 HS and E19 HSTS vs. E19 HS single-cell transcriptome datasets. Through the integration of a curated ligand–receptor interaction database, cell-type-specific communication patterns across the two breeds at corresponding developmental stages were characterized.

**Figure 3:**
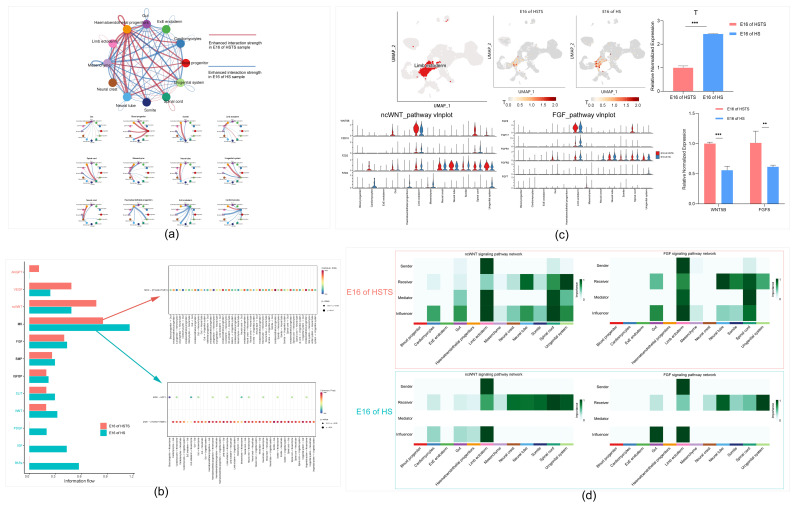
Single-cell transcriptomic atlas of E16 and E19 HSTS and HS embryos. (**a**) Intercellular communication probability network of E16 HSTS and E16 HS samples. (**b**) Information flow and MK pathway heatmap of E16 HSTS and E16 HS samples. (**c**) *T* gene expression in E16 HSTS and E16 HS samples and expressions of *WNT5B* and *FGF8* in the ncWNT and FGF pathways (** *p* < 0.01, *** *p* < 0.001). (**d**) The roles of ncWNT and FGF pathways in E16 HSTS and E16 HS samples.

The results revealed that in the E16 HSTS sample, 11 distinct cell populations participated in intercellular interactions, whereas in the E16 HS sample, all 12 cell populations were involved (Figure 3a). Statistical analysis indicated that the *MDK-ITGA6+ITGB1* signaling axis showed enriched communication probability in both groups (Figure 3b). Meanwhile, breed-specific differences were observed in *T* gene activity: although *T* was highly expressed in the AER of the E16 HS sample, its canonical downstream targets, *WNT5B* and *FGF8*, exhibited stronger expression in the AER of the E16 HSTS sample (Figure 3c).

Further role analysis of *T*-specific pathways (noncanonical WNT and FGF signaling) within the AER demonstrated distinct regulatory architectures. In the E16 HSTS sample, the AER may coordinate embryonic development through a potential multidirectional ncWNT-FGF feedback network, whereas in the E16 HS sample, the AER primarily mediated regulation through a linear signaling cascade (Figure 3d).

**Figure 4:**
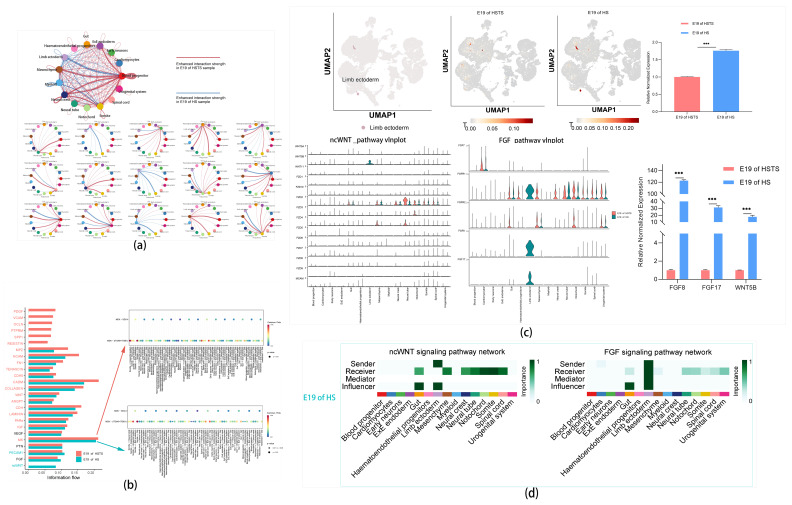
CellChat analysis of E19 HSTS and E19 HS samples. (**a**) Network diagram of intercellular interaction probabilities between E19 HSTS and E19 HS samples. (**b**) Information flow and MK pathway heatmap of E19 HSTS and E19 HS samples. (**c**) *T* gene expression in E19 HSTS and E19 HS samples and expression of *WNT5B*, *FGF8*, and *FGF17* within the ncWNT and FGF pathways (*** *p* < 0.001). (**d**) The roles of the ncWNT and FGF pathways in E19 HS samples.

At the E19 stage, CellChat analysis revealed that all 15 identified cell populations participated in intercellular communication in both the HSTS and HS samples (Figure 4a). Quantitative assessment of ligand–receptor interactions demonstrated that the *MDK_ITGA6*+*ITGB1* signaling axis exhibited the strongest communication capacity across both groups (Figure 4b). *T* expression was markedly reduced in the E19 HSTS sample, whereas in the E19 HS sample, *T* was specifically enriched in the AER. Moreover, *T* displayed spatial co-localization with its canonical downstream targets *WNT5B* and *FGF8*, showing highly consistent expression patterns (Figure 4c). Pathway role analysis within *T*-specific AER populations further uncovered breed-dependent regulatory architectures. In the AER of the E19 HS sample, the FGF signaling pathway functioned predominantly through cell-autonomous regulation, whereas the ncWNT pathway mediated cross-cellular coordination by targeting distal cell populations through paracrine signaling (Figure 4d). These breed-specific differences in cell–cell communication, particularly the loss of AER-associated FGF and ncWNT signaling in HSTS, may explain the divergent tail development between the two breeds.

### 3.4. Pseudotime Analysis of Cross-Stage Developmental Regulation

Pseudotime analysis enables the ordering of individual cells along a developmental trajectory based on sequential gene expression dynamics. However, pseudotime reconstructions are mathematical inferences based on only two time points (E16 and E19); they should be interpreted as a continuum rather than actual developmental time. Validation with intermediate time points (e.g., E17, E18) is needed. Monocle (v2.22.0) was adopted for the pseudotime analysis of E16 vs. E19 samples in both HSTS and HS.

**Figure 5:**
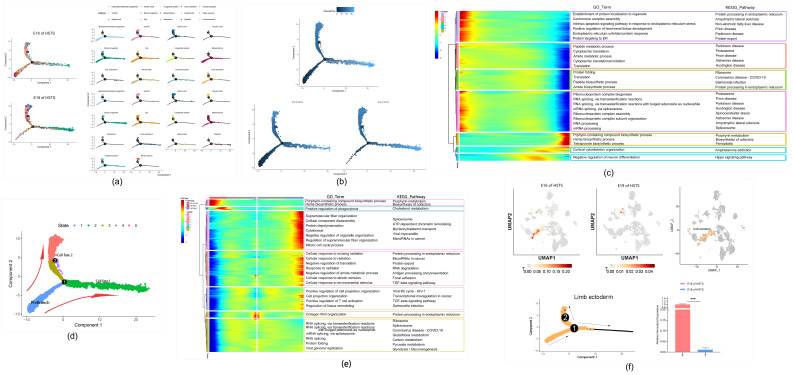
Pseudotime trajectory analysis of E16 and E19 HSTS samples. (**a**) Pseudotime developmental trajectories of distinct cell populations in E16 and E19 HSTS samples. (**b**) Pseudotime developmental trajectories of E16 and E19 HSTS samples. (**c**) Heatmap of DEGs along the pseudotime trajectory in E16 vs. E19 HSTS samples, with corresponding GO and KEGG enrichment analyses. (**d**) Distribution of pseudotime branch points between E16 and E19 HSTS samples. (**e**) Heatmap of branch-specific DEGs in E16 vs. E19 HSTS samples, with corresponding GO and KEGG enrichment analyses. (**f**) Temporal expression features of the *T* gene in E16 and E19 HSTS samples, with experimental validation (*** *p* < 0.001).

The results of E16 vs. E19 HSTS are depicted in Figure 5a. Based on developmental biology principles, the lower-left region of the coordinate map was designated as the developmental origin (Figure 5b). Differentially expressed genes (DEGs) identified along the pseudotime axis were subjected to GO and KEGG enrichment analysis (k = 7; only GO biological process terms are displayed). The results suggested a sequential transition in gene function: from fundamental cellular construction (Clusters 1–2), through tissue- and organ-specific developmental regulation (neurodevelopmental regulation, Clusters 3–5), and finally to highly specialized cellular functions (hematopoietic activity, Cluster 7). This provides a model of the regulatory network underlying hepatic development between E16 and E19 (Figure 5c).

To further resolve branching dynamics, Branched Expression Analysis Modeling (BEAM) was conducted using the R package clusterProfiler3 (v4.2.2). This approach constructs branch-point models (BranchPoints) to systematically identify DEGs across divergent developmental trajectories. At BranchPoint1, the reconstructed topology suggested three distinct states: Pre-branch (State 3) represented progenitor cell status, Cell fate 1 (State 2) mapped to one differentiation pathway, and Cell fate 2 (State 1) corresponded to an alternative trajectory (Figure 5d). Subsequent GO and KEGG enrichment analyses of BranchPoint1 DEGs (k = 7; only BP terms are shown in this figure) jointly validated the core functions of each gene cluster from the biological process and signaling pathway levels. Clusters 1, 2, and 5 emerged as drivers of Cell fate 2, while Clusters 4 and 6 were enriched in hematopoietic programs of Cell fate 1. Clusters 3 and 7 occupied more fundamental or upstream positions, providing structural and regulatory prerequisites for subsequent lineage specification (Figure 5e).

In addition, the distribution of AER cells with *T* gene-specific expression was mapped along the pseudotime trajectory. AER was detectable only in E16 HSTS and absent in E19 HSTS (Figure 5f). Within the HSTS embryonic samples, *T* expression gradually decreased over pseudotime, concomitant with the progressive reduction in AER cells.

**Figure 6:**
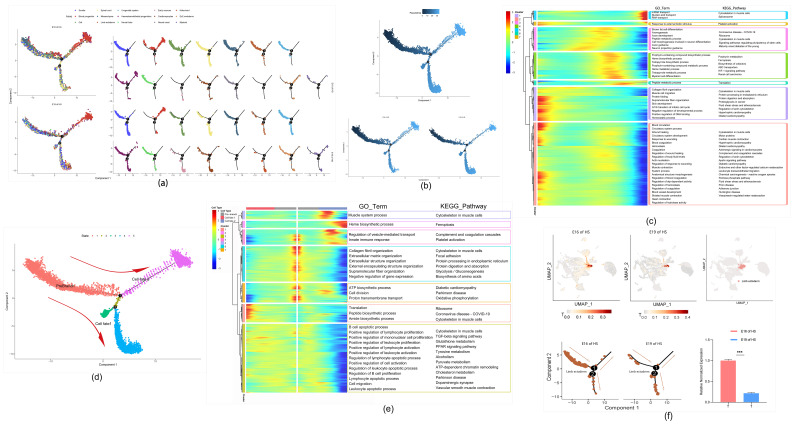
Pseudotime analysis of E16 and E19 HS samples. (**a**) Pseudotime developmental trajectories of individual cell populations in E16 and E19 HS samples. (**b**) Overall pseudotime trajectories of E16 and E19 HS samples. (**c**) Heatmap and corresponding GO and KEGG enrichment analyses of pseudotime-distributed DEGs in E16 and E19 HS samples. (**d**) Branchpoint distribution along pseudotime trajectories of E16 and E19 HS samples. (**e**) Heatmap of pseudotime-distributed DEGs and associated GO/KEGG enrichment analyses in E16 and E19 HS samples. (**f**) Temporal expression dynamics and validation of the *T* gene in E16 and E19 HS samples (*** *p* < 0.001).

The pseudotime trajectories of cell populations in E16 HS versus E19 HS are shown in Figure 6a. Based on developmental biology, the upper-left corner of the coordinate plot was designated as the developmental origin. In the E16 HS sample, as development progressed to day 19, the number of cells at the trajectory origin markedly decreased, whereas the number of cells at the distal ends of the two branches increased substantially (Figure 6b).

DEGs along the pseudotime trajectory were subsequently subjected to GO and KEGG enrichment analyses (k = 7; only GO biological process terms are shown). The enrichment results suggested a highly coordinated developmental program along the trajectory, transitioning from early tissue construction (ECM organization, cell proliferation) → system development (circulatory and nervous systems) → cell differentiation (erythroid lineage) → terminal functional maturation (RNA regulation, myofibrillar and skeletal processes) (Figure 6c).

Pseudotime BEAM analysis was then performed to further resolve branch-specific regulatory programs. At BranchPoint1, Pre-branch corresponded to State1, Cell fate 1 encompassed States 2, 3, and 4, and Cell fate 2 corresponded to State5 (Figure 6d). GO and KEGG enrichment of DEGs at BranchPoint1 (k = 7; only BP terms displayed) provided complementary functional insights, outlining a detailed regulatory map: the Pre-branch region (Clusters 6 and 7) was primarily involved in preparatory functions for differentiation, including structural assembly (cytoskeleton and cell adhesion) and energy metabolism; the Cell fate 1 trajectory exhibited functional enrichment in immune regulation (Cluster 3) and protein synthesis (Cluster 1); the Cell fate 2 trajectory displayed clear myogenic signatures (Cluster 5) and terminal heme metabolism (Cluster 2), whereas Cluster 4 may mediate shared processes between the two lineages, such as immune-related functions (Figure 6e).

Additionally, the distribution of *T* gene-specific AER cells along the developmental trajectory was illustrated. *T* gene expression was restricted to the AER population and declined significantly in parallel with the reduction in AER cells observed at day 19 (Figure 6f). The pseudotime trajectories indicate that HSTS embryos undergo accelerated differentiation, which may compromise proper tail outgrowth. The accelerated differentiation trajectory observed in HSTS embryos is consistent with premature depletion of progenitor populations, which may limit tail outgrowth and contribute to the short tail. However, these computational inferences require experimental validation.

### 3.5. Analysis of T Gene Transcription Factors

To delve into the regulatory mechanisms underlying the short-tail phenotype, the transcriptional regulatory function of the *T* gene was analyzed. Single-cell analysis of transcription factors and their regulatory networks can reveal cell-type-specific and state-dependent gene expression patterns, providing critical insights into complex biological processes, such as development, differentiation, and disease pathogenesis. SCENIC is a computational framework designed to infer gene regulatory networks and associated cell states from scRNA-seq data. By analyzing the expression patterns and regulatory targets of transcription factors, SCENIC elucidates the biological basis of cellular heterogeneity.

*T* exhibits cell-type-specific expression in AER cells. However, in the E19 HSTS sample, no AER population was annotated, and *T* expression levels were extremely low. Consequently, conventional regulon analysis (significance threshold NES ≥ 1.5) failed to detect the *T* regulon. Even when the NES threshold was relaxed to a non-standard level (NES ≥ 1.0), the *T* regulon remained undetectable in E19 HSTS. However, this relaxed threshold is not recommended for regulon inference and may increase false positives; therefore, this result should be interpreted with caution. All main conclusions regarding regulon activity are based on the standard NES ≥ 2.5 threshold. Therefore, the analysis was restricted to samples in which *T* regulons were detectable (E16 HSTS vs. E16 HS and E16 HS vs. E19 HS).

**Figure 7:**
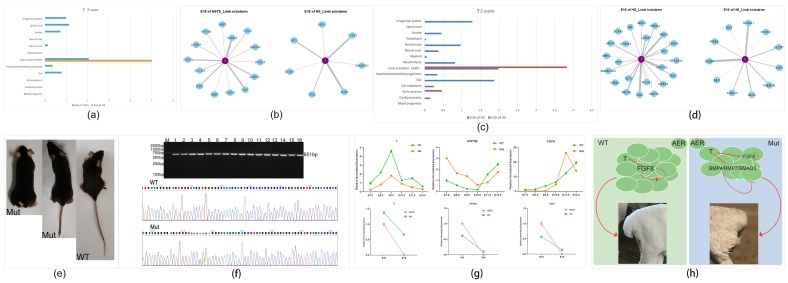
*T* gene mutation leads to short-tail phenotype: transcriptional activity, target genes, expression dynamics, and mechanistic model. (**a**) Z-score profiles of *T* transcription factor activity in E16 HSTS and E16 HS samples. (**b**) Downstream target genes of the *T* gene in the AER of E16 HSTS and E16 HS samples. (**c**) Z-score profiles of *T* transcription factor activity in E16 HS and E19 HS samples. (**d**) Downstream target genes of the *T* gene in the AER of E16 HS and E19 HS samples. (**e**) Tail morphological comparison between wild-type and mutant mice carrying the *T* gene mutation. (**f**) Genotyping results of mutant mice (M: DL2000 Plus; 1–8: wild-type mouse *T* gene amplification products; 9–16: mutant mouse *T* gene amplification products). (**g**) Temporal expression patterns of *T*, *WNT5B*, and *FGF8* in wild-type and mutant mouse embryos, as well as in HSTS and HS embryos. Data are presented as mean ± SD (n = 3 biological replicates per group). Exact *p*-values and Cohen’s d effect sizes are provided in Section 3.5. (**h**) Schematic representation of the mechanism by which *T* gene mutation leads to the short-tail phenotype.

Using pySCENIC (v0.11.2), *T* transcription factor activity was assessed across these samples. Regulon Specificity Scores (RSS) were calculated and Z-score normalized. In the E16 HSTS vs. E16 HS group, AER cells in both samples exhibited high Z-scores. However, in the E16 HS sample, *T* expression was spatially restricted to the AER, whereas in the E16 HSTS sample, *T* exhibited broader regulatory activity (Figure 7a). High-confidence target genes (IM ≥ 0.95) were identified based on importance metrics (IM). In E16 HSTS, 14 high-confidence targets were detected, twice the number found in E16 HS (7 targets) (Figure 7b).

In both the E16 and E19 HS samples, AER cells exhibited prominent *T* activation. For E16 HS, Z-scores were confined to two discrete regions and peaked in the AER, whereas in E19 HS, broader regulatory activity was observed, with the maximal Z-score remaining localized within the AER (Figure 7c). Applying IM ≥ 0.95, 21 high-confidence *T* targets were identified in E16 AER cells, compared with 11 targets at E19 (Figure 7d).

The author has demonstrated that the short-tail phenotype in sheep is associated with the c.G334T mutation, and a corresponding CRISPR/Cas9 mouse model carrying this mutation was generated (Figure 7e). In this mouse model, only heterozygous mutants were viable, as homozygous *T* gene mutants resulted in embryonic lethality at E10.5. Heterozygous mutant mice exhibited mid- and short-tail phenotypes. Short-tail mice displayed a tail bud of 0.5–1.5 cm above the anus, and mid-tail mice had blunt tail tips compared with the conical wild-type tails (Figure 7e). This model was used to validate *T* transcription factor expression patterns observed in scRNA-seq of HSTS and HS early embryos.

Since the model only existed in the heterozygous state, both wild-type and mutant individuals were present within the same litter. To ensure accurate genotyping of the samples, genomic DNA from newborn mouse toes was subjected to PCR (Figure 7f). The PCR products were analyzed by 1.5% agarose gel electrophoresis, yielding bands within the 500–700 bp range, consistent with the expected product size (651 bp). Sanger sequencing revealed a single peak in wild-type mice (190–220 bp region: …CCTGGGGGCAAA…), whereas mutant mice showed characteristic double peaks corresponding to the c.G334T mutation (…CCT(A)G(T)GGGGCAAA…).

RT-qPCR analysis of *T*, *WNT5B*, and *FGF8* expression in wild-type and mutant mice, as well as sheep embryos, demonstrated that mutant *T* expression was consistently lower than wild-type across all time points in both species. In sheep, HSTS had lower *T* expression than HS at both E16 and E19. In mouse, mutant *T* was lower than wild-type from E7 to E12. *WNT5B* showed a U-shaped expression curve in mouse mutants (higher before E10.5, lower thereafter), whereas in sheep it was consistently lower in HSTS at both stages. *FGF8* transiently increased at E11.5 in mouse mutants but continuously decreased in sheep mutants. These species-specific differences are discussed further in Section 4.5. Suggesting that *FGF8* may respond to AER dynamics and potentially participate in feedback regulation of its regression. Collectively, these findings indicate that the *T* gene mutation perturbs the spatiotemporal expression of key developmental genes, most notably altering the expression trajectories of *FGF8* and *WNT5B* (Figure 7g).

Statistical analysis revealed: For sheep *T* at E16, HSTS expressed 1.000 ± 0.035 (mean ± SD) vs. HS 1.375 ± 0.004 (*p* < 0.001, Cohen’s d = 15.2); at E19, HSTS 0.0134 ± 0.0017 vs. HS 0.668 ± 0.0038 (*p* < 0.001, d = 229). For sheep *WNT5B* at E16, HSTS 1.002 ± 0.008 vs. HS 0.611 ± 0.009 (*p* < 0.001, d = 46.1); at E19, HSTS 0.00195 ± 0.00005 vs. HS 0.104 ± 0.018 (*p* < 0.001, d = 8.0). For sheep *FGF8* at E16, HSTS 1.003 ± 0.082 vs. HS 0.569 ± 0.009 (*p* < 0.001, d = 7.6); at E19, HSTS 0.00398 ± 0.00009 vs. HS 0.136 ± 0.024 (*p* < 0.001, d = 7.7). For mouse *T* at E10.5, Mut 0.917 ± 0.048 vs. WT 1.290 ± 0.033 (*p* < 0.001, d = 8.9); at E11.5, Mut 0.508 ± 0.006 vs. WT 1.544 ± 0.040 (*p* < 0.001, d = 36.5). For mouse *WNT5B* at E10.5, Mut 0.590 ± 0.008 vs. WT 0.161 ± 0.004 (*p* < 0.001, d = 69.5); at E11.5, Mut 0.826 ± 0.009 vs. WT 1.544 ± 0.040 (*p* < 0.001, d = 25.0). For mouse *FGF8*, the values were identical to *WNT5B* at both time points.

Despite reduced *T* expression, the c.G334T mutation was associated with an increased number of high-confidence target genes in E16 HSTS compared to E16 HS. This difference may reflect altered DNA-binding properties of the mutant *T* protein, but this interpretation is computational and requires functional validation (e.g., ChIP-seq). The mutation was located in the DNA-binding domain, likely modifying binding affinity or specificity and reshaping the regulatory network. In E16 HSTS, the *T* mutant showed transcriptomic signatures of increased expression of BMP pathway genes (*BMP4*, *BMP7*, *SMAD3*) and decreased expression of AER marker *FGF8*. BMP-SMAD signaling is known to promote programmed apoptosis, and reduced *FGF8* expression may compromise AER survival. These transcriptional changes are consistent with accelerated AER regression. However, this model is based solely on transcriptomic correlations; direct experimental validation (e.g., pharmacological inhibition of BMP signaling in tail explants or genetic crosses in mice) is required to establish causality. Conversely, sustained FGF8 expression in wild-type facilitated normal tail development. In summary, the c.G334T mutation disrupts the transcriptional regulatory network via altered DNA-binding properties, ultimately inducing the short-tail phenotype (Figure 7h). The combination of reduced *T* expression, expanded regulon target genes, and altered *FGF8*/*WNT5B* dynamics suggests that the c.G334T mutation disrupts the transcriptional network controlling AER maintenance, leading to premature AER regression and a short tail. Transcriptomic data indicate AER regression, without direct morphological proof.

## 4. Discussion

The adaptive evolution of species and the underlying genetic regulatory mechanisms during embryogenesis remain central topics in developmental biology. This study employed single-cell multi-omics approaches to delineate breed-specific developmental programs in early sheep embryos, with a particular emphasis on the mechanistic basis of the short-tail phenotype in HSTS, providing new insights into how a single nucleotide mutation affects tail development. By integrating cross-breed (HSTS vs. HS), cross-timepoint (E16 vs. E19), and cross-species (sheep vs. mouse) datasets, this study interrogated two central issues: (1) how environmental adaptation drives divergence in metabolism and intercellular communication, and (2) how the *T* gene c.G334T mutation reshapes gene regulatory networks to trigger AER apoptosis, thereby affecting tail development. The findings provide new insights into vertebrate tail development and identify potential genetic targets for improving stress-resilient traits in livestock.

### 4.1. Differences in Cell–Cell Communication Networks at E16

At E16, we detected 12 cell types in both breeds, broadly similar to a previous sheep embryo atlas [47], although relative proportions varied. This variation likely reflects differences in reference databases or annotation methods, but it does not affect the main conclusions about cell–cell communication. Cell–cell communication analysis revealed that the MDK_ITGA6+ITGB1 signaling axis broadly mediates angiogenesis and tissue remodeling in both breeds, aligning with known roles of *MDK* in angiogenesis and tissue remodeling. Nevertheless, breed-specific regulatory modules were observed: in HSTS, cardiomyocytes reinforced cardiovascular development via *VEGFB_VEGFR1* and *ANGPT1_TEK*, whereas HS maintained metabolic homeostasis through *IGF2_IGF2R/IGF1R*, consistent with known imprinting regulatory mechanisms.

Activation of *IGFBP3_TMEM219* was detected in HSTS, potentially promoting apoptosis. Notably, HSTS urogenital system cells engaged in cross-tissue communication via *WNT5B_FZD2* and BMP pathways [48]. In conjunction with the c.G334T mutation, this likely attenuates regulation of *WNT5B* and *FGF8*, consistent with the genetic analyses of Zhi et al. [3] and Yang et al. [49]. At the single-cell level, this regulatory attenuation triggers compensatory high expression within the AER [50,51], thereby driving the establishment of an accelerated developmental strategy in HSTS centered on ncWNT/FGF multidirectional feedback, in sharp contrast to the relatively linear signaling in HS. While previous studies have described individual pathways (e.g., *VEGFB* in cardiovascular development or *IGF2* imprinting in metabolic homeostasis), our single-cell data reveal that these pathways are differentially active in specific cell types across breeds, suggesting that breed-specific adaptations may be driven by cell-type-specific signaling rewiring rather than global pathway changes. These breed-specific signaling differences, particularly the accelerated and multidirectional ncWNT/FGF feedback in HSTS versus linear signaling in HS, may predispose HSTS to faster AER regression and consequently a shorter tail.

### 4.2. Evolution of Signaling Networks at E19 and the Implications of AER Loss

AER cells were detected only in HS. Their absence in HSTS is consistent with premature AER regression, which may be influenced by the *T* gene mutation. This contrasts with the gradual post-limb-patterning AER regression described by Verheyden and Sun [52], suggesting that the *T* mutation accelerates this process.

Intercellular communication analysis revealed that *MDK_ITGA6+ITGB1* remained a dominant signaling axis, consistent with known roles of *MDK*, and echoing the E16 observation of *MDK*-centered interactions. HSTS cardiomyocytes regulated myocardial differentiation via *COL4A2* and *NCAM1_FGFR1*, while HS extraembryonic endoderm cells may enhance signaling efficiency through receptor clustering.

Unlike Gros et al. [53], HS AER integrated multiple pathways—including *MDK*, *FN1*, coagulation, and fibrinolysis—to sustain AER function. Intestinal cells in HSTS modulated notochord morphology via *FN1_ITGAV+ITGB1* and basement membrane remodeling, whereas HS relied more on *MDK* dual-function modules. Moreover, HSTS vascular endothelial progenitors engaged an additional mechanosensory pathway through *VCAM1–ITGA9+ITGB1*, whereas HS maintained endothelial barrier integrity via *PECAM1*.

These results indicate that, although core signaling pathways are conserved, the regulatory details and cell-type-specificity differ markedly between breeds. In particular, AER-associated FGF and ncWNT pathways remain active in HS but are functionally lost in HSTS due to AER absence, potentially representing a critical mechanism underlying divergent tail development. The functional loss of AER-associated FGF and ncWNT signaling in HSTS directly correlates with the absence of AER cells at E19, providing a mechanistic link between the *T* mutation and the short-tail phenotype. The observation that HSTS lack active AER-related FGF and ncWNT signaling while HS retain them highlights a potential mechanistic link between the *T* mutation and tail length. However, direct causality remains to be tested; it is possible that other pathways (e.g., Notch, Hedgehog) also contribute to the phenotypic divergence, as suggested by studies in other vertebrates. Whether the tail length difference directly confers adaptive advantage in cold versus warm climates remains a hypothesis that requires further testing (e.g., through physiological experiments or comparative genomics across more breeds.

### 4.3. Temporal Regulatory Mechanisms of HSTS Embryo Development from E16 to E19

Pseudotime analysis revealed a shift from early progenitor states at E16 to terminally differentiated cell types at E19, with stage-specific gene expression modules. This transition suggests that HSTS embryos undergo accelerated differentiation, which may limit tail outgrowth.

Branch-point analysis indicated that cell clusters at Branchpoint 1 exhibit distinct functional specializations. Pre-branch cells primarily contribute to tissue construction and energy supply; Cell fate1 is biased toward neural differentiation, whereas Cell fate2 is enriched for muscle and hematopoietic lineages. Specifically, Cluster 1 maintains genomic stability via XRCC5, SMC1A, supporting prior conclusions by Bladen and Musio [54,55]; Cluster 2 regulates cytoskeletal architecture and metabolic homeostasis, consistent with Peng and Zaffagnini [56,57]; Cluster 5 is enriched for genes associated with neural synapses and immune tolerance, aligning with enrichment of synaptic and immune-related genes in this cluster; however, the functional significance of immune-associated genes in tail development remains unclear.; Cluster 6 exhibits high expression of *AHSG* and *TTR*, indicative of multi-organ progenitor characteristics, consistent with Dabrowska [58]. KEGG analysis further suggested that TGF-β and chromatin remodeling pathways in Cluster 1 coordinately regulate mesoderm differentiation, supporting the views of Liu and Crosswhite [59,60]; in Cluster 5, crosstalk between Wnt signaling and pluripotency networks aligns with the mechanistic insights of Sonavane et al. [61]. Collectively, these findings delineate the molecular basis for the orderly differentiation of HS embryos from a pluripotent state to functionally specialized lineages.

*T* gene expression is significantly downregulated from E16 to E19, coinciding with AER developmental regression, consistent with Verheyden and Sun [52]. AER degeneration may involve feedback-mediated suppression of *T* expression via FGF/Wnt signaling, a mechanism that warrants validation through spatial transcriptomics.

While pseudotime analysis provides a temporal ordering of transcriptional states, it does not directly measure developmental time. The inferred trajectories are based on only two sampling points and therefore represent a simplified view of continuous development. Experimental validation with additional time points is required. Experimental approaches, such as in vivo lineage tracing or the inclusion of additional embryonic stages (e.g., E17, E18), are needed to directly validate the inferred trajectories. Although our pseudotime analysis aligns with published gene-expression dynamics during somitogenesis and neurogenesis, the inferred trajectories are based on only two time points and lack spatial resolution. Therefore, these results should be interpreted as a model rather than a definitive timeline.

### 4.4. Notochord Dynamics and Lineage Specification in HS Embryos

Lineage progression in HS embryos followed a predictable sequence from mesenchymal progenitors to somite, neuronal, and notochord lineages, consistent with classical descriptions of vertebrate development [62]. Pseudotime analysis further outlined a temporal order of gene expression changes from early structural processes to late functional specialization.

Branch-point analysis further highlighted distinct lineage trajectories. Cluster 6 contributes structural support via collagen fibers, consistent with Sun et al. [63]; Cluster 7 exhibits high metabolic activity to sustain proliferation; Cluster 3 is biased toward the neural lineage, with key genes promoting neurogenesis through zinc homeostasis and associated signaling pathways, in agreement with enrichment of neurogenesis-related genes; Cluster 4 facilitates angiogenesis via platelet activation and the complement system, in line with known roles of platelet activation and complement in angiogenesis (though direct evidence in tail development is lacking); Cluster 5 predominantly regulates cardiac development, enriched for myogenic pathways, consistent with cytoskeletal regulation mechanisms proposed by Lim and Plachta [64]; Cluster 2 is oriented toward erythroid differentiation. Overall, Pre-branch cells primarily perform foundational functions, whereas post-branch trajectories bifurcate into two distinct fates: one toward neural differentiation, the other toward myogenic and hematopoietic specialization, illustrating the orderly transition from a pluripotent state to multiple functional lineages.

Furthermore, the *T* gene was found to be highly expressed in the AER at E16 but downregulated by E19 concomitant with the reduction in AER cells, consistent with the classical description of progressive AER regression after limb patterning by Verheyden and Sun [52]. This contrasts with the premature AER disappearance observed in HSTS, underscoring the pivotal role of the *T* gene mutation in temporal developmental regulation. While these lineage trajectories are informative for general embryogenesis, their direct relevance to tail development lies in the timely coordination of mesodermal and neural crest derivatives; any disruption in this coordination could affect tail elongation.

### 4.5. T Gene Mutation Reshapes Regulatory Networks to Affect AER Survival

The analyses of scRNA-seq and SCENIC revealed that the *T* gene c.G334T mutation reduces its expression level, consistent with the reports of Zhi et al. [3] and Yang et al. [49]. Notably, the mutation also markedly expands the activity range of its regulon and increases the number of high-confidence target genes. This reduced expression is accompanied by an increased number of high-confidence target genes, raising the possibility that the mutation may alter the DNA-binding properties of the *T* protein and thereby reshape its regulatory network. Functional experiments are required to test this hypothesis.

Specifically, in line with Fernandez-Teran and Lewandoski [65,66], the mutation may weaken *T* protein binding to the FGF8 promoter while simultaneously enhancing regulation of the BMP4/7-SMAD3 apoptosis pathway. These transcriptional changes suggest a model in which BMP-SMAD signaling is upregulated and FGF8 is downregulated, potentially leading to accelerated AER cell death. We infer that the combined effects of reduced FGF8 expression and increased BMP pathway activity may contribute to premature AER degeneration and the short-tail phenotype. It must be emphasized that this model is speculative and derived solely from transcriptomic data. Direct functional testing (e.g., AER-specific manipulation of BMP or FGF signaling in vivo) is required to determine causality. The proposed BMP-FGF antagonism specifically affects AER maintenance, a structure essential for tail elongation. Thus, the transcriptional changes observed in HSTS provide a plausible explanation for the short tail. These inferences are derived from transcriptional data and should be considered hypothesis-generating. Functional perturbation experiments (e.g., BMP inhibitor treatment in embryonic tail cultures, or conditional knockout of Bmp4 in the AER) are needed to directly test the proposed BMP/FGF antagonism. Until such functional experiments are performed, the proposed model remains a hypothesis rather than an established mechanism.

Although our data point to BMP/FGF imbalance as a plausible cause of AER regression, alternative mechanisms cannot be excluded. For instance, the *T* mutation might also affect notochord extension, somite formation, or tail bud mesenchyme proliferation independently of the AER. Moreover, changes in cell adhesion, migration, or extracellular matrix composition could contribute to tail shortening. Our single-cell data do not directly rule out these possibilities, and future studies should investigate whether additional pathways (e.g., Wnt/β-catenin, retinoic acid signaling) are involved.

In wild-type HS embryos, *T* gene activity exhibits clear temporal dynamics: at E16, it supports AER function through regulation of targets such as *SLIT2*, consistent with metabolic reprogramming observed in rapidly proliferating cells; by E19, *T* activity progressively declines in parallel with AER regression, aligning with the transcriptional network convergence described by Dudley et al. [67].

Cross-species comparison reveals partial conservation of *T* downregulation in both species. However, the dynamics of *WNT5B* and *FGF8* differ: *WNT5B* exhibits a U-shaped curve in mouse but a monotonic decrease in sheep; *FGF8* shows a transient peak at E11.5 in mouse mutants but a steady decline in sheep mutants. These differences may reflect species-specific developmental timing, genetic compensation, or differences in AER regression kinetics. Therefore, while the mouse model supports a role for the *T* mutation in perturbing *WNT5B* and *FGF8* expression, direct extrapolation to sheep should be made with caution (see Section 3.5). These findings are partially consistent with previous observations in mouse models [51].

A notable species difference is that the homozygous *T* c.G334T mutation causes embryonic lethality in mice (by E10.5) but not in sheep, where homozygous individuals are viable. This discrepancy may reflect species-specific genetic compensation or differences in embryonic development timing. Therefore, while the mouse model supports key transcriptional changes, results should be interpreted with caution when extrapolating to sheep.

### 4.6. Study Limitations

We acknowledge that direct histological or morphological evidence of AER degeneration was not obtained in this study. Our conclusion is therefore based on transcriptomic signatures, including the absence of AER cell clusters in E19 HSTS, downregulation of AER marker genes (*FGF8* and *WNT5B*), and activation of pro-apoptotic BMP signaling.

Additionally, the sample size for sheep embryos in this study is relatively small (one or three biological replicates per group). This is due to the difficulty of obtaining staged embryos from large animals, the high cost of single-cell sequencing, and the rarity of specific genotypes. The limited number of sheep embryos remains a constraint, and the results should be interpreted with caution.

To partially overcome these limitations, key findings (e.g., expression dynamics of *T*, *WNT5B*, and *FGF8*) were independently validated by RT-qPCR using multiple biological replicates in both sheep and a CRISPR/Cas9 mouse model carrying the same *T* c.G334T mutation (n > 3 per genotype and time point). The cross-species consistency supports the robustness of our main conclusions. Species differences in the phenotypic outcome of the homozygous mutation (lethal in mice, viable in sheep) highlight the need for caution when extrapolating mouse data to sheep. Additionally, the proposed BMP/FGF regulatory model has not been experimentally validated and remains a hypothesis based on transcriptomic evidence.

Future studies combining single-cell transcriptomics with sectioning and staining of tail bud tissues, as well as larger sample sizes in sheep, are warranted to directly confirm AER regression and further solidify the mechanistic model. A limitation of our scRNA-seq analysis is that multiple testing correction was not applied. However, the main conclusions are based on a small set of pre-selected, biologically relevant genes that were further validated by RT-qPCR, which minimizes the risk of false positives.

## 5. Conclusions

This study constructed single-cell transcriptomic maps of HSTS and HS embryos, revealing breed-specific adaptive differentiation patterns: developmental progression follows a “undifferentiated (E16) → terminally specialized (E19)” trajectory, with MDK_ITGA6+ITGB1 as a central intercellular communication hub. Mechanistically, the *T* gene c.G334T mutation is associated with transcriptomic changes suggestive of BMP-SMAD pathway activation and *FGF8* suppression, which may contribute to premature AER regression. BMP and FGF pathway involvement is supported by scRNA-seq differential expression (*BMP4*, *BMP7*, *SMAD3*, *FGF8*). However, direct links between these molecular changes and environmental adaptation remain speculative and require further investigation. Cross-species validation in a mouse model supports key transcriptional changes, but species differences (lethality in mice versus viability in sheep) caution against direct extrapolation. Additionally, due to the small sample size of scRNA-seq, these conclusions are primarily descriptive for the analyzed embryos and should be validated in larger cohorts before generalization to other populations.

## Figures and Tables

**Table 1:** Genotyping Primer sequence (mouse).

Primer	Sequence (5′ → 3′)	Gene ID	Product Length
T	Forward P: CAAAGATGCTTGCGAGAC	20997	651 bp
	Reverse P: GGAACCAATGCTCACCTAT		

**Table 2:** RT-qPCR primer sequences.

Primer	Sequence (5′ → 3′)	Gene ID	Product Length
*GAPDH* (sheep)	Forward P: CGGCACAGTCAAGGCAGAGAAC	443005	219 bp
	Reverse P: CACGTACTCAGCACCAGCATCAC		
*T* (sheep)	Forward P: CACCAAGAACGGCAGGAGGATG	101114280	122 bp
	Reverse P: CGTACTTCCAGCGGTGGTTGTC		
*FGF8* (sheep)	Forward P: TCAGCCGCCGTCTCATCCG	101113716	150 bp
	Reverse P: TGCTCCCGAAGGTGTCTGTCTC		
*FGF17* (sheep)	Forward P: GGAGACAGACACATTCGGAAGCC Reverse P: ACAATCTCGGTGAACACGCAGTC	101118502	135 bp
*WNT5B* (sheep)	Forward P: TCCTGGTGGTCCTTGGCGATG	101121415	135 bp
	Reverse P: CATGTGCTCCTGGTAGAGTTGGC		
*GAPDH* (mouse)	Forward P: GGTGAAGGTCGGTGTGAACG	14433	233 bp
	Reverse P: CTCGCTCCTGGAAGATGGTG		
*T* (mouse)	Forward P: AACAGCTCTCCAACCTATGC	20997	244 bp
	Reverse P: AGCCTCGAAAGAACTGAGC		
*WNT5B* (mouse)	Forward P: TCCCGGACAGTCTAGAGACCC	22419	265 bp
	Reverse P: GGGGAAAGCCCAGGAAGTTG		
*FGF8* (mouse)	Forward P: CTCCAAGCCCAGGAAGGC	14179	266 bp
	Reverse P: TCTTCTGCCATGGCGTTGAT		

**Table 3:** Genotyping of the *T* Gene at the c.334 Locus in HSTS and HS (Yellow: missense mutation; Gray: no mutation).

Number	The Genotype of the c.334 Locus in the *T* Gene
HSTS1 (ram)	GGTGCCCTGGGGCAAGCCAGAGCCG
HSTS1 (ewe)	GGTGCCCTGGGGCAAGCCAGAGCCG
HSTS2 (ewe)	GGTGCCCTGGGGCAAGCCAGAGCCG
HSTS3 (ewe)	GGTGCCCTGGGGCAAGCCAGAGCCG
HSTS4 (ewe)	GGTGCCCTGGGGCAAGCCAGAGCCG
HSTS5 (ewe)	GGTGCCCTGGGGCAAGCCAGAGCCG
HSTS6 (ewe)	GGTGCCCTGGGGCAAGCCAGAGCCG
HSTS7 (ewe)	GGTGCCCTGGGGCAAGCCAGAGCCG
HSTS8 (ewe)	GGTGCCCTGGGGCAAGCCAGAGCCG
HSTS9 (ewe)	GGTGCCCTGGGGCAAGCCAGAGCCG
HSTS10 (ewe)	GGTGCCCTGGGGCAAGCCAGAGCCG
HS1 (ram)	GGTGCCGGGGGGCAAGCCAGAGCCG
HS1 (ewe)	GGTGCCGGGGGGCAAGCCAGAGCCG
HS2 (ewe)	GGTGCCGGGGGGCAAGCCAGAGCCG
HS3 (ewe)	GGTGCCGGGGGGCAAGCCAGAGCCG
HS4 (ewe)	GGTGCCGGGGGGCAAGCCAGAGCCG
HS5 (ewe)	GGTGCCGGGGGGCAAGCCAGAGCCG
HS6 (ewe)	GGTGCCGGGGGGCAAGCCAGAGCCG
HS7 (ewe)	GGTGCCGGGGGGCAAGCCAGAGCCG
HS8 (ewe)	GGTGCCGGGGGGCAAGCCAGAGCCG
HS9 (ewe)	GGTGCCGGGGGGCAAGCCAGAGCCG
HS10 (ewe)	GGTGCCGGGGGGCAAGCCAGAGCCG

## Data Availability

Single-cell RNA sequencing is available from the Gene Expression Omnibus (GEO) database with the accession number GSE308927.

## Abbreviations

The following abbreviations are used in this manuscript:

AER	Apical Ectodermal Ridge
BP	Biological Process
DEGs	Differentially expressed genes
E16	Embryo at 16 days
E19	Embryo at 19 days
ECM	Extracellular Matrix
GEO	Gene Expression Omnibus
GO	Gene Ontology
HS	Hu sheep
HSTS	Hulun Buir short-tailed sheep
IM	Importance Measure
KEGG	Kyoto Encyclopedia of Genes and Genomes
MDK	Midkine
Mut	Mutant
ncWNT	Non-canonical WNT Signaling Pathway
PCR	Polymerase chain reaction
PMSG	Pregnant Mare Serum Gonadotropin
RT-qPCR	Real-Time Quantitative Reverse Transcription PCR
scRNA-seq	Single-cell RNA sequencing
TFs	Transcription Factors
WT	Wild Type

## Appendix A

### Appendix A.1

**Table A1:** The quality control results of the single-cell sequencing of the samples in this study.

Samples	E16 of HSTS	E16 of HS	E19 of HSTS	E19 of HS
Estimated Number of Cells	10,709	12,645	11,114	12,064
Mean Reads per Cell	44,382	34,557	36,311	28,783
Median Genes per Cell	2054	1906	1598	1504
Number of Reads	475,283,103	436,971,211	403,564,895	347,235,149
Q30 Bases in RNA Read	93.40%	91.70%	93.00%	93.10%
Reads Mapped to Genome	97.00%	95.90%	96.60%	96.80%
Reads Mapped Confidently to Genome	88.80%	87.40%	85.50%	86.50%
Reads Mapped Confidently to Intergenic Regions	20.60%	18.50%	14.20%	15.60%
Reads Mapped Confidently to Exonic Regions	57.50%	53.50%	57.20%	56.60%
Reads Mapped Confidently to Transcriptome	51.20%	47.10%	49.30%	49.50%
Fraction Reads in Cells	82.80%	91.00%	94.30%	94.90%
Average reads detected per cell	44,382	34,557	36,311	28,783
Detected total genes (at least one UMI)	17,415	18,352	18,199	18,070
Median number of UMIs per cell	10,644	8564	7926	6440

### Appendix A.2

**Table A2:** List of key parameters and software versions used in scRNA-seq analysis.

Step/Parameter	Value/Setting	Software/Version
Data filtering		
nFeature_RNA range	500–6000	Seurat (v4.1.1)
nCount_RNA range	1000–30,000	Seurat (v4.1.1)
percent.mt threshold (high-quality cells)	<5%	Seurat (v4.1.1)
Additional retention criteria	>200 genes & <10% mitochondrial content	Seurat (v4.1.1)
Normalization		
Method	LogNormalize, scale factor = 10,000	Seurat (v4.1.1)
Imputation	MAGIC, ‘genes = “all_genes”’	MAGIC (v3.0.0)
Highly variable gene selection		
Method	‘FindVariableFeatures’, ‘selection.method = “vst”’	Seurat (v4.1.1)
Number of features	2000	Seurat (v4.1.1)
Scaling and regression		
Variables to regress	‘percent.mt’	Seurat (v4.1.1)
Dimensionality reduction (PCA)		
Number of principal components (PCs)	First 25 PCs	Seurat (v4.1.1)
Batch effect correction		
Method	Harmony, batch defined by sample source	Harmony (v0.1.0)
Dimensions for Harmony	First 25 PCs	–
Clustering (after Harmony)		
Neighbor graph dimensions	‘dims = 1:25’	Seurat (v4.1.1)
Clustering resolution (initial)	0.2 (for Harmony clustering)	Seurat (v4.1.1)
Clustering resolution (final)	0.25	Seurat (v4.1.1)
Visualization		
UMAP dimensions	‘dims = 1:25’	Seurat (v4.1.1)
Differential expression analysis		
Test	Wilcoxon rank-sum test	Seurat (v4.1.1)
‘only.pos’	TRUE	Seurat (v4.1.1)
*p*-value threshold	<0.05	Seurat (v4.1.1)
Ranking metric	avg_log2FC	Seurat (v4.1.1)
Gene enrichment analysis		
Tools	clusterProfiler (GO, KEGG)	clusterProfiler (v4.2.2)

## References

[1] Kalds P., Zhou S., Gao Y., Cai B., Huang S., Chen Y., Wang X. (2022). Genetics of the phenotypic evolution in sheep: A molecular look at diversity-driving genes. Genet. Sel. Evol..

[2] Su H., Chen L., Yang G., Wang D.Q., Li X.N., Song Y.L., Cao G.F. (2024). The Transcriptional Landscape of the Early Embryonic Developmental Biology in Hulunbuir Short-Tailed Sheep. Indian J. Anim. Res..

[3] Zhi D., Da L., Liu M., Cheng C., Zhang Y., Wang X., Li X., Tian Z., Yang Y., He T. (2018). Whole Genome Sequencing of Hulunbuir Short-Tailed Sheep for Identifying Candidate Genes Related to the Short-Tail Phenotype. G3.

[4] Su H., Zhi D., Song Y., Yang Y., Wang D., Li X., Cao G. (2024). Exploring the formation mechanism of short-tailed phenotypes in animals using mutant mice with the *TBXT* gene c.G334T developed by CRISPR/Cas9. Gene.

[5] Kamme F., Salunga R., Yu J., Tran D.T., Zhu J., Luo L., Bittner A., Guo H.Q., Miller N., Wan J. (2003). Single-cell microarray analysis in hippocampus CA1: Demonstration and validation of cellular heterogeneity. J. Neurosci..

[6] Pijuan-Sala B., Griffiths J.A., Guibentif C., Hiscock T.W., Jawaid W., Calero-Nieto F.J., Mulas C., Ibarra-Soria X., Tyser R.C.V., Ho D.L.L. (2019). A single-cell molecular map of mouse gastrulation and early organogenesis. Nature.

[7] Cao J., Spielmann M., Qiu X., Huang X., Ibrahim D.M., Hill A.J., Zhang F., Mundlos S., Christiansen L., Steemers F.J. (2019). The single-cell transcriptional landscape of mammalian organogenesis. Nature.

[8] Soldatov R., Kaucka M., Kastriti M.E., Petersen J., Chontorotzea T., Englmaier L., Akkuratova N., Yang Y., Häring M., Dyachuk V. (2019). Spatiotemporal structure of cell fate decisions in murine neural crest. Science.

[9] Negretti N.M., Plosa E.J., Benjamin J.T., Schuler B.A., Habermann A.C., Jetter C.S., Gulleman P., Bunn C., Hackett A.N., Ransom M. (2021). A single-cell atlas of mouse lung development. Development.

[10] Cui Y., Zheng Y., Liu X., Yan L., Fan X., Yong J., Hu Y., Dong J., Li Q., Wu X. (2019). Single-Cell Transcriptome Analysis Maps the Developmental Track of the Human Heart. Cell Rep..

[11] Cao J., O’Day D.R., Pliner H.A., Kingsley P.D., Deng M., Daza R.M., Zager M.A., Aldinger K.A., Blecher-Gonen R., Zhang F. (2020). A human cell atlas of fetal gene expression. Science.

[12] Tyser R.C.V., Mahammadov E., Nakanoh S., Vallier L., Scialdone A., Srinivas S. (2021). Single-cell transcriptomic characterization of a gastrulating human embryo. Nature.

[13] Braun E., Danan-Gotthold M., Borm L.E., Lee K.W., Vinsland E., Lönnerberg P., Hu L., Li X., He X., Andrusivová Ž. (2023). Comprehensive cell atlas of the first-trimester developing human brain. Science.

[14] Stuart T., Butler A., Hoffman P., Hafemeister C., Papalexi E., Mauck W.M., Hao Y., Stoeckius M., Smibert P., Satija R. (2019). Comprehensive Integration of Single-Cell Data. Cell.

[15] van Dijk D., Sharma R., Nainys J., Yim K., Kathail P., Carr A.J., Burdziak C., Moon K.R., Chaffer C.L., Pattabiraman D. (2018). Recovering Gene Interactions from Single-Cell Data Using Data Diffusion. Cell.

[16] Korsunsky I., Millard N., Fan J., Slowikowski K., Zhang F., Wei K., Baglaenko Y., Brenner M., Loh P.R., Raychaudhuri S. (2019). Fast, sensitive and accurate integration of single-cell data with Harmony. Nat. Methods.

[17] Qiu X., Mao Q., Tang Y., Wang L., Chawla R., Pliner H.A., Trapnell C. (2017). Reversed graph embedding resolves complex single-cell trajectories. Nat. Methods.

[18] Hubaud A., Pourquié O. (2014). Signalling dynamics in vertebrate segmentation. Nat. Rev. Mol. Cell Biol..

[19] Oates A.C., Morelli L.G., Ares S. (2012). Patterning embryos with oscillations: Structure, function and dynamics of the vertebrate segmentation clock. Development.

[20] van den Brink S.C., Alemany A., van Batenburg V., Moris N., Blotenburg M., Vivié J., Baillie-Johnson P., Nichols J., Sonnen K.F., Martinez Arias A. (2020). Single-cell and spatial transcriptomics reveal somitogenesis in gastruloids. Nature.

[21] Han X., Zhou Z., Fei L., Sun H., Wang R., Chen Y., Chen H., Wang J., Tang H., Ge W. (2020). Construction of a human cell landscape at single-cell level. Nature.

[22] Binagui-Casas A., Dias A., Guillot C., Metzis V., Saunders D. (2021). Building consensus in neuromesodermal research: Current advances and future biomedical perspectives. Curr. Opin. Cell Biol..

[23] Dady A., Davidson L., Halley P.A., Storey K.G. (2022). Human spinal cord in vitro differentiation pace is initially maintained in heterologous embryonic environments. eLife.

[24] Zhao L., Song W., Chen Y.G. (2022). Mesenchymal-epithelial interaction regulates gastrointestinal tract development in mouse embryos. Cell Rep..

[25] Plein A., Fantin A., Denti L., Pollard J.W., Ruhrberg C. (2018). Erythro-myeloid progenitors contribute endothelial cells to blood vessels. Nature.

[26] Jiang Z., Lu Z., Kou S., Feng T., Wei Y., Gao Z., Deng D., Meng J., Lin C.P., Zhou B. (2021). Overexpression of Kdr in adult endocardium induces endocardial neovascularization and improves heart function after myocardial infarction. Cell Res..

[27] Iruela-Arispe M.L. (2018). A dual origin for blood vessels. Nature.

[28] Groves J.A., Gillman C., DeLay C.N., Kroll T.T. (2019). Identification of Novel Binding Partners for Transcription Factor *Emx2*. Protein J..

[29] Li H., Jakobson M., Ola R., Gui Y., Kumar A., Sipilä P., Sariola H., Kuure S., Andressoo J.O. (2019). Development of the urogenital system is regulated via the 3’UTR of *GDNF*. Sci. Rep..

[30] Muntean C., Chirtes C., Baczoni B., Banescu C. (2023). PAX2 Gene Mutation in Pediatric Renal Disorders-A Narrative Review. Int. J. Mol. Sci..

[31] Filimonow K., de la Fuente R. (2022). Specification and role of extraembryonic endoderm lineages in the periimplantation mouse embryo. Theriogenology.

[32] Amadei G., Handford C.E., Qiu C., De Jonghe J., Greenfeld H., Tran M., Martin B.K., Chen D.Y., Aguilera-Castrejon A., Hanna J.H. (2022). Embryo model completes gastrulation to neurulation and organogenesis. Nature.

[33] Frank D., Yusuf Rangrez A., Friedrich C., Dittmann S., Stallmeyer B., Yadav P., Bernt A., Schulze-Bahr E., Borlepawar A., Zimmermann W.H. (2019). Cardiac *α-Actin* (*ACTC1*) Gene Mutation Causes Atrial-Septal Defects Associated with Late-Onset Dilated Cardiomyopathy. Circ. Genom. Precis. Med..

[34] Li I.M.H., Horwell A.L., Chu G., de Crombrugghe B., Bou-Gharios G. (2017). Characterization of Mesenchymal-Fibroblast Cells Using the Col1a2 Promoter/Enhancer. Methods Mol. Biol..

[35] Herrera-Quiterio G.A., Encarnación-Guevara S. (2023). The transmembrane proteins (TMEM) and their role in cell proliferation, migration, invasion, and epithelial-mesenchymal transition in cancer. Front. Oncol..

[36] Boulet A.M., Moon A.M., Arenkiel B.R., Capecchi M.R. (2004). The roles of *Fgf4* and *Fgf8* in limb bud initiation and outgrowth. Dev. Biol..

[37] Lau K., Tao H., Liu H., Wen J., Sturgeon K., Sorfazlian N., Lazic S., Burrows J.T., Wong M.D., Li D. (2015). Anisotropic stress orients remodelling of mammalian limb bud ectoderm. Nat. Cell Biol..

[38] Martin A., Maher S., Summerhurst K., Davidson D., Murphy P. (2012). Differential deployment of paralogous *Wnt* genes in the mouse and chick embryo during development. Evol. Dev..

[39] Pshennikova E.S., Voronina A.S. (2019). Neural Crest-An Unusual Population of Embryonic Cells. Mol. Biol..

[40] Kaufmann L.T., Gierl M.S., Niehrs C. (2011). *Gadd45a*, *Gadd45b* and *Gadd45g* expression during mouse embryonic development. Gene Expr. Patterns.

[41] Tian T., Liu J., Lu X., Qiu X., Wei J., Wang C., Liu M., Yin S., Jin L., Wang L. (2022). Selenium protects against the likelihood of fetal neural tube defects partly via the arginine metabolic pathway. Clin. Nutr..

[42] Peng Z., Ren Z., Tong Z., Zhu Y., Zhu Y., Hu K. (2023). Interactions between *MFAP5* + fibroblasts and tumor-infiltrating myeloid cells shape the malignant microenvironment of colorectal cancer. J. Transl. Med..

[43] Li Z., Zhou B., Zhu X., Yang F., Jin K., Dai J., Zhu Y., Song X., Jiang G. (2023). Differentiation-related genes in tumor-associated macrophages as potential prognostic biomarkers in non-small cell lung cancer. Front. Immunol..

[44] Jang Y., Oh S., Hall A.J., Zhang Z., Tropea T.F., Chen-Plotkin A., Rosenthal L.S., Dawson T.M., Na C.H., Pantelyat A.Y. (2024). Biomarker discovery in progressive supranuclear palsy from human cerebrospinal fluid. Clin. Proteom..

[45] Sainio M.T., Ylikallio E., Mäenpää L., Lahtela J., Mattila P., Auranen M., Palmio J., Tyynismaa H. (2018). Absence of *NEFL* in patient-specific neurons in early-onset Charcot-Marie-Tooth neuropathy. Neurol. Genet..

[46] Klar A., Jessell T.M., Ruiz i Altaba A. (1992). Control of floor plate identity and function in the embryonic nervous system. Cold Spring Harb. Symp. Quant. Biol..

[47] He T., Guo W., Yang G., Su H., Dou A., Chen L., Ma T., Su J., Liu M., Su B. (2023). A Single-Cell Atlas of an Early Mongolian Sheep Embryo. Vet. Sci..

[48] Hadas R., Rubinstein H., Mittnenzweig M., Mayshar Y., Ben-Yair R., Cheng S., Aguilera-Castrejon A., Reines N., Orenbuch A.H., Lifshitz A. (2024). Temporal *BMP4* effects on mouse embryonic and extraembryonic development. Nature.

[49] Yang G., Wang C., Su H., Wang D., Dou A., Chen L., Ma T., Liu M., Su J., Xu X. (2022). Development and Application of a High-Resolution Melting Analysis with Unlabeled Probes for the Screening of Short-Tailed Sheep *TBXT* Heterozygotes. Animals.

[50] Yamaguchi T.P., Bradley A., McMahon A.P., Jones S. (1999). A Wnt5a pathway underlies outgrowth of multiple structures in the vertebrate embryo. Development.

[51] Lolas M., Valenzuela P.D., Tjian R., Liu Z. (2014). Charting *Brachyury*-mediated developmental pathways during early mouse embryogenesis. Proc. Natl. Acad. Sci. USA.

[52] Verheyden J.M., Sun X. (2017). Embryology meets molecular biology: Deciphering the apical ectodermal ridge. Dev. Biol..

[53] Gros J., Hu J.K., Vinegoni C., Feruglio P.F., Weissleder R., Tabin C.J. (2010). *WNT5A*/JNK and FGF/MAPK pathways regulate the cellular events shaping the vertebrate limb bud. Curr. Biol..

[54] Bladen C.L., Lam W.K., Dynan W.S., Kozlowski D.J. (2005). DNA damage response and Ku80 function in the vertebrate embryo. Nucleic Acids Res..

[55] Musio A. (2020). The multiple facets of the *SMC1A* gene. Gene.

[56] Peng H., Qiao J., Wang G., Shi W., Xia F., Qiao R., Dong B. (2023). A collagen-rich arch in the urochordate notochord coordinates cell shaping and multi-tissue elongation. Curr. Biol..

[57] Zaffagnini G., Cheng S., Salzer M.C., Pernaute B., Duran J.M., Irimia M., Schuh M., Böke E. (2024). Mouse oocytes sequester aggregated proteins in degradative super-organelles. Cell.

[58] Dabrowska A.M., Tarach J.S., Wojtysiak-Duma B., Duma D. (2015). *Fetuin-A* (*AHSG*) and its usefulness in clinical practice. Review of the literature. Biomed. Pap. Med. Fac. Univ. Palacky. Olomouc Czech Repub..

[59] Liu C., Peng G., Jing N. (2018). TGF-β signaling pathway in early mouse development and embryonic stem cells. Acta Biochim. Biophys. Sin..

[60] Crosswhite P.L. (2019). ATP-dependent chromatin remodeling complexes in embryonic vascular development and hypertension. Am. J. Physiol. Heart Circ. Physiol..

[61] Sonavane P., Willert K. (2023). Wnt signaling and the regulation of pluripotency. Curr. Top. Dev. Biol..

[62] Bianco P., Robey P.G., Simmons P.J. (2008). Mesenchymal stem cells: Revisiting history, concepts, and assays. Cell Stem Cell.

[63] Sun M., Luo E.Y., Adams S.M., Adams T., Ye Y., Shetye S.S., Soslowsky L.J., Birk D.E. (2020). Collagen XI regulates the acquisition of collagen fibril structure, organization and functional properties in tendon. Matrix Biol..

[64] Lim H.Y.G., Plachta N. (2021). Cytoskeletal control of early mammalian development. Nat. Rev. Mol. Cell Biol..

[65] Fernandez-Teran M., Ros M.A. (2008). The Apical Ectodermal Ridge: Morphological aspects and signaling pathways. Int. J. Dev. Biol..

[66] Lewandoski M., Sun X., Martin G.R. (2000). *Fgf8* signalling from the AER is essential for normal limb development. Nat. Genet..

[67] Dudley A.T., Ros M.A., Tabin C.J. (2002). A re-examination of proximodistal patterning during vertebrate limb development. Nature.

